# Quarante cas de cryptococcose neuroméningée diagnostiqués en 21 ans au laboratoire de parasitologie de l’hôpital Ibn Sina de Rabat

**DOI:** 10.11604/pamj.2019.33.249.18011

**Published:** 2019-07-23

**Authors:** Fatima-Zahra Bandadi, Chaimae Raiss, Aziza Moustachi, Mohamed Lyagoubi, Sara Aoufi

**Affiliations:** 1 Laboratoire Central de Parasitologie-Mycologie Médicale, Centre Hospitalier Ibn Sina de Rabat, Rue Lamfadel Cherkaoui, BP 6527, Rabat, Maroc; 2 Faculté de Médecine et de Pharmacie, Université Mohamed V, Avenue Mohammed Belarabi Elalaoui, BP 6203 Rabat-Instituts, Rabat, Maroc

**Keywords:** Cryptococcose, VIH, immunodépression, Maroc, Cryptococcosis, HIV, immunosuppression, Morocco

## Abstract

La cryptococcose neuroméningée (CNM) est une mycose opportuniste fréquente et sévère causée par une levure encapsulée *Cryptococcus neoformans.* Elle est fréquente chez l'immunodéprimé en particulier le sujet atteint du virus de l'immunodéficience humaine (VIH) à un stade avancé de la maladie, elle est rare chez l'immunocompétent. Nous rapportons 40 cas de cryptococcose neuroméningée (CNM) diagnostiqués au laboratoire de parasitologie de l'hôpital Ibn Sina de Rabat sur une période de 21 ans (1993 à 2014). Le diagnostic de la cryptococcose neuroméningée a reposé sur la mise en évidence de *Cryptococcus neoformans* dans le liquide céphalorachidien (LCR) après l'examen direct à l'encre de Chine et la culture sur milieux de sabouraud sans actidione, ainsi que la recherche d'antigènes solubles cryptococciques. Trente cinq patients étaient infectés par le VIH, deux patients étaient apparemment immunocompétents et 3 patients immunodéprimés non VIH (30 hommes et 10 femmes). L'âge moyen des patients était de 38 ans. La cryptococcose neuroméningée était révélatrice de l'infection par le VIH dans 13 cas. Dans 22 cas elle a représenté une complication du SIDA. Vingt sept patients de notre série ont été traités par une monothérapie à base de fluconazole. L'amphotéricine B a été utilisée chez 13 patients. L'évolution a été favorable pour 13 patients (32.5%) et trois cas ont connu une complication (7.5%). Alors que 18 patients sont décédés (45%) et 6 ont été perdus de vue (15%). Pour un diagnostic rapide, la recherche de *Cryptococcus neoformons* doit être appliquée systématiquement devant le moindre signe neurologique.

## Abstract (en)

Neuromeningeal cryptococcosis is a common and severe opportunistic fungal infection caused by the encapsulated yeast Cryptococcus neoformans. It commonly occurs in immunocompromised patients, in particular in subjects with advanced stage HIV while it is rare in immunocompetent patients. We report 40 cases of neuromeningeal cryptococcosis (NMC) diagnosed at the Mycology-Parasitology Department of the Ibn Sina hospital in Rabat, over a 21-year period (1993-2014). The diagnosis was based on nested-PCR-based assay for the detection of Cryptococcus neoformans after staining with China ink and culture on Sabouraud agar without actidione as well as on the identification of soluble cryptococcal antigens. Thirty-five patients had HIV infection, 2 patients were apparently immunocompetent and 3 were immunocompromised patients without HIV (30 men and 10 women). The average age of patients was 38 years; neuromeningeal cryptococcosis was indicative of HIV infection in 13 cases. In 22 cases it was a complication of AIDS. Twenty-seven patients of our series were treated with fluconazole monotherapy. Amphotericin B was used in 13 patients. Outcome was favorable in 13 patients (32.5%) while 3 patients had complications (7.5%). Eighteen patients died (45%) and 6 were lost to follow-up (15%). The tests to diagnose a Cryptococcus neoformans infection should be performed systematically in patients with neurological signs for early diagnosis.

## Introduction

La cryptococcose représente après la toxoplasmose la deuxième infection opportuniste du système nerveux central chez les sidéens. Son incidence dans les pays occidentaux a significativement diminué depuis la généralisation des thérapies anti-rétrovirales. Malgré la bonne prise en charge des malades atteints du VIH, des cas sporadiques de cryptococcose neuroméningée restent repérés chez les sidéens. Ce travail décrit les aspects épidémiologiques, cliniques et biologiques de cette mycose dans le contexte marocain à travers une série de 40 cas sur une période de 21 ans.

## Méthodes

Entre les années 1993 et 2014 (21ans), le laboratoire de parasitologie-mycologie de l'hôpital Ibn Sina de Rabat a isolé 40 cas de cryptococcose neuroméningée. Tous les renseignements ont été tirés des registres du laboratoire de parasitologie et des dossiers des patients hospitalisés dans les services. Au niveau de chaque dossier, nous avons relevé: l'âge, le sexe, le terrain d'immunodépression, les données cliniques, biologiques et thérapeutiques ainsi que l'évolution clinique des patients. Le diagnostic de la cryptococcose neuroméningée a reposé sur la mise en évidence des levures encapsulées dans le LCR par l'examen direct à l'encre de Chine. Ce champignon a été identifié après culture sur les milieux sabouraud sans actidione à partir des galeries d'identification Auxacolor^TM^. La recherche d'antigènes circulants de *Cryptococcus neoformans* dans le LCR a été réalisée par la technique d'agglutination au latex. La trousse utilisée était Pastorex^TM^ Crypto plus. Aucune étude sur les espèces et les variétés de cryptocoque n'a été faite au Maroc . Tous les cas décrits de cryptococcose auraient probablement pour agent responsible *Cryptococus neoformans.*

## Résultats

**Figure 1:**
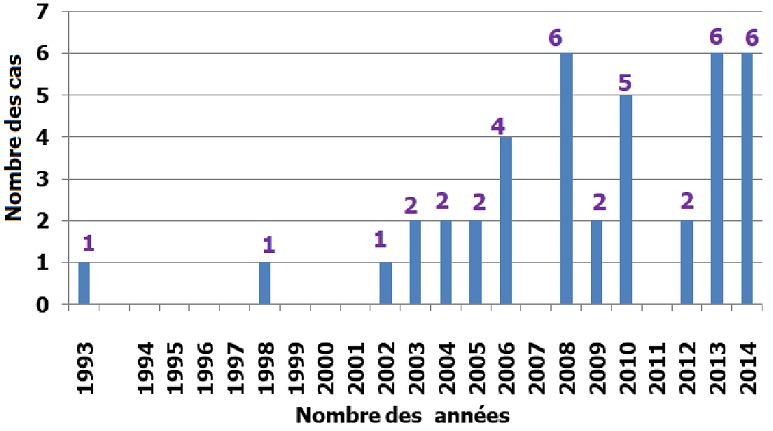
Nombre de cas de cryptococcose en fonction des années

Cette étude est étalée sur une période de 21 ans de janvier 1993 à décembre 2014. D'après les résultats (Figure 1), nous constatons que pendant les dix premières années (1993-2002), un faible nombre de cas est enregistré (3 cas), soit en moyenne un cas tous les 3-4 ans. La deuxième décennie à partir de 2003 est caractérisée par une évolution très importante des cas (37 cas) soit en moyenne 3 cas par an. En passant de 2 cas en 2003 à 4 cas en 2006 pour atteindre un maximum de cas en 2008, 2013 et 2014 avec six cas. Le sexe masculin était prédominant, 30 hommes contre seulement 10 femmes avec un sexe ratio de 3. L'âge moyen était de 38 ans avec des extrêmes de 24 ans et de 65 ans. Dans notre série, 35 patients étaient séropositifs pour le VIH et cinq patients était séronégatifs; deux patients étaient sous corticothérapie, deux cas de cryptococcose ont été rapportés chez deux patients où aucun facteur d'immunodépression n'a été retrouvé et un cas de grossesse a été enregistré. Sur 35 cas de patients séropositifs au VIH, la cryptococcose a compliqué le SIDA dans 22 cas, tandis qu'elle a révélé ce stade dans 13 cas.

Dans notre série aucun cas de syndrome de reconstitution immune n'a été trouvé. Les signes cliniques de la cryptococcose étaient dominés essentiellement par la fièvre, les céphalées et la raideur de la nuque. Parmi les malades, sept avaient une infection opportuniste associée. Trois cas de toxoplasmose, deux cas de pneumocystose et deux cas de candidose œsophagienne. Dix sept patients ont développé une cryptococcose extra méningée. Sept avaient une fongémie, dont un patient a présenté des lésions cutanées, cinq avaient une atteinte pulmonaire et deux seulement avaient une localisation urinaire. Le cryptocoque a aussi colonisé le tube digestif dans trois cas. Parmi les 40 cas de cryptococcose, un seul patient a présenté une hypertension intracrânienne pour lequel une ponction lombaire décompressives a été réalisée. L'étude de l'immunité cellulaire a été focalisée sur la numération des lymphocytes T CD4, réalisée chez 26 patients. Dix-sept patients avaient un taux de CD4<100 éléments/mm^3^ montrant que leur immunodépression étaient très détériorée voire avancée au moment du diagnostic. Le LCR avait un aspect clair dans les 40 cas de La cryptococcose neuroméningée. Vingt quatre patients ont bénéficié d'une analyse cytochimique du LCR. Une hyperalbuminorachie a été observé chez 15 patients et 13 patients ont présenté une hypoglycorachie.

L'examen direct à l'encre de Chine était positif à 100% pour toute la série et a montré la présence de levures encapsulées. La culture du LCR était positive dans tous les cas et a permis l'isolement et l'identification de *Cryptococus neoformans.* La recherche d'antigénes solubles cryptococcique dans le LCR a été réalisée chez 36 patients et elle était positive dans tous les cas. La bithérapie à base de L'amphotéricine B+5 flucytosine n'a pas été adoptée dans notre série. Vingt sept patients ont été traités par une monothérapie à base de fluconazole (400mg/j). L'amphotéricine B (1mg/kg/j) a été utilisée chez 13 patients dont 4 ont bénéficié d'un relais par le fluconazole (400mg/j). Parmi les 40 cas de cryptococcose l'évolution était favorable pour 13 patients dont trois cas ont été traités par l'amphotéricine B avec relais par le fluconazole, 18 patients sont décédés; six patients traités par une monothérapie par l'amphotéricine B (1mg/kg/j) et cinq patients traités par le fluconazole seul 400mg/j, alors que trois cas ont connu une rechute et une complication de leur état. Six perdus de vue ont été enregistrés.

## Discussion

Au Maroc peu d'études ont été menées sur la prévalence et l'incidence de la CNM, seulement des cas sporadiques qui sont rapportés: 14 cas colligés de 1987 à 1998 au CHU Ibn Rochd de Casablanca [1], et 43 cas diagnostiqués au même centre sur une période de 5 ans du 1^er^ janvier 2010 au 30 juin 2015 [2]. 9 cas ont été diagnostiqués au CHU de Rabat [3]. La cryptococcose est souvent plus observée chez l'homme que chez la femme. La majorité des études parcourues [4, 5] confirment cette prédominance qui résulte de la fréquence de l'infection à VIH chez le sexe masculin. La tranche d'âge juvénile est la plus active sexuellement. Elle prédomine dans de nombreuses études [4, 5]. Dans notre série l'âge moyen des patients était de 38 ans. La majorité des cas de cryptococcose survient chez des patients infectés par le VIH. Dans notre étude, 35 patients ont développé une cryptococcose sur un terrain d'immunodépression lié a ce virus. Cette infection opportuniste survient souvent au stade tardif du SIDA avec des taux de CD4 généralement inférieur à 100 cellules/mm^3^, où le risque de survenue de l'infection est multiplié par 8 [1]. Ceci se voit clairement dans notre série, puisque pour 17 patients, nous avons relevé des taux de CD4<100 éléments/mm^3^. Au Maroc, le nombre de cas de VIH/Sida cumulés pendant la période 1986-2015 est: 11.298 cas dont 6271 cas de SIDA et 4.949 de patients séropositifs et 78 cas non renseignés de stade [6].

Dans le cadre de l'infection par le VIH, l'atteinte neuroméningée reste la localisation la plus fréquente. Néanmoins le champignon peut atteindre tous les organes lors de l'infection disséminée, surtout avec des atteinte pluri systémiques. Cette dissémination est associée à un pronostic péjoratif [7]. Sur les 40 cas de cryptococcose neuroméningée, cinq patients étaient séronégatifs au VIH: la corticothérapie est reconnue comme facteur favorisant indépendant de la maladie associée à une mortalité accrue [7]: deux patients étaient sous corticothérapie dans notre série. La grossesse est un autre facteur de risque pour le développement de la cryptococcoses [8]: nous avons relevé un cas d'une patiente enceinte sans aucun facteur d'immunodépression et qui a développé une méningoencéphalite à cryptocoque. Cette mycose survient également chez des patients apparemment indemnes de toute immunodépression. De rares publications sur des terrains immunocompétents sont faites [9]: deux cas de cryptococcose ont été rapportés dans notre série, chez deux patients chez lesquels aucun facteur d'immunodépression n'a été retrouvé.

Le LCR était le plus souvent clair à prédominance lymphocytaire, avec une hyperalbuminorachie chez 15 patients et une hypoglycorachie chez 13 patients. Nos résultats sont semblables à ceux rapportés par la pluspart des auteurs [10]. Le traitement de référence de la cryptococcose neuroméningée comporte une bithérapie associant l'amphotéricine B à raison de 1mg/kg/j par voie intraveineuse et la 5-flucytosine à 100mg/kg/j per os en quatre prise. Ce protocole thérapeutique n'a pas été adopté dans notre contexte du fait de la non disponibilité des médicaments et de leur coût élevé, ce qui a conduit à une monothérapie non recommandée au fluconazole à des doses de 400mg/j, administré chez 27 patients et à l'amphotéricine B chez 13 patients dont 4 ont bénéficié d'un relais par le fluconazole. Le taux de mortalité observé (18 cas/40 cas) soit 45% est comparable à celui retrouvé dans d'autres études [11, 12].

## Conclusion

La cryptococcose neuroméningée reste une mycose profonde opportuniste et grave, qui survient lors des stades avancés de l'immunodépression par le VIH, mais également chez des patients séronégatifs à ce virus. La réduction de la mortalité par cette mycose impose la nécessité d'un diagnostic rapide et d'un traitement approprié par le respect de l'utilisation du protocole recommandé.

### État des connaissances actuelles sur le sujet

- La cryptococcose est une mycose systémique opportuniste fréquente et sévère;
- Elle est fréquente chez l'immunodéprimé et rare chez l'immunocompétent;
- C'est une affection habituellement mortelle au cours du SIDA.

### Contribution de notre étude à la connaissance

- Enrichir nos connaissances sur cette maladie sur le plan épidémiologiques, clinique et biologique dans le contexte marocain vu que les études menées sur cette maladie sont rare au Maroc.

## Conflits des intérêts

Les auteurs ne déclarent aucun conflit d'intérêts.
